# Genome Sequence of the *Bacillus subtilis* Biofilm-Forming Transformable Strain PS216

**DOI:** 10.1128/genomeA.00288-13

**Published:** 2013-06-20

**Authors:** Russell Durrett, Mathieu Miras, Nicolas Mirouze, Apurva Narechania, Ines Mandic-Mulec, David Dubnau

**Affiliations:** Institute for Computational Biomedicine, Weill Medical College of Cornell University, New York, New York, USAa; Public Health Research Institute, New Jersey Medical School, Newark, New Jersey, USAb; Université de Toulouse (Toulouse III), Toulouse, Francec; UMR1319 Micalis, Bat. Biotechnologie (440), INRA, Domaine de Vilvert, Jouy-en-Josas, Franced; Sackler Institute for Comparative Genomics, American Museum of Natural History, New York, New York, USAe; Biotechnical Faculty, University of Ljubljana, Ljubljana, Sloveniaf

## Abstract

*Bacillus subtilis* PS216, a strain isolated in Slovenia, has been sequenced. PS216 is transformable and forms robust biofilms, making it useful for the study of competence regulation in an undomesticated bacterium.

## GENOME ANNOUNCEMENT

*Bacillus subtilis* is the most-studied Gram-positive model organism (1). It has become apparent that the standard reference strain *B. subtilis* 168 has been modified by decades of inadvertent selection in the laboratory, thereby acquiring a high frequency of transformability and losing the ability to form biofilms (2). Recently, “undomesticated” strains, notably *B. subtilis* NCIB3610, have been investigated intensively because of their ability to form robust biofilms (2). However, NCIB3610 is poorly transformable, limiting its usefulness for the study of genetic competence and compromising its ability to be manipulated genetically. *B. subtilis* PS216, which was isolated in Slovenia from sandy soil, forms robust biofilms and is more transformable than NCIB3610. Based on phylogenetic analysis of three concatenated protein-coding genes (*dnaJ*, *gyrA*, and *rpoB*), PS216 is most closely related to *B. subtilis* subsp. *subtilis* and belongs to a clade demarcated as the putative ecotype 10 (3). Strain PS216 resides in the same quorum-sensing pherotype group as 168 (4, 5).

The genome sequence of *B. subtilis* PS216 was generated as described in Koren et al. (6). Briefly, 274 Mb of PacBio long-read data were error corrected with 150 bp MiSeq data using the pacBioToCA pipeline, resulting in approximately 71 Mb of corrected long reads that were then assembled by the Celera assembler. This assembly contained 146 contigs, 90% of the assembly being in 26 contigs that are >42 kb.

The initial assembly yielded a total of 112 single-nucleotide changes compared to the reference strain 168 (accession no. NC_000964) and 140 single-nucleotide polymorphisms (SNPs) compared to strain NCIB3610 (accession no. NZ_CM000488). SNPs were identified in SAMtools (7) using short read alignments generated by BWA (8). Sequencing of PCR products confirmed four of the nucleotide changes in genes of interest (*oppD*, *comP*, *degQ*, *sigH*). Of the 112 nucleotides that differed between strains 168 and PS216, 27 were identical in sequence between PS216 and NCIB3610. These include the confirmed nucleotide changes in *degQ*, *oppD*, and *sigH*.

Notably, no large plasmids were detected in PS216, such as the one present in NCIB3610 (2). We used NUCmer (9) and ABACAS (10) to order and orient the contigs with respect to the reference, an analysis which revealed that both the 20,521-bp integrative and conjugative element (ICEBs1) (11) and the 134,385-bp SPβ temperate bacteriophage present in 168 (12) were missing from PS216. The absence of the latter two elements was verified by sequencing a PCR product that crossed the two insertion sites.

We anticipate that this sequence information for PS216 will facilitate comparative studies of the development and physiology in *Bacillus* species.

### Nucleotide sequence accession numbers.

This Whole-Genome Shotgun project has been deposited at DDBJ/EMBL/GenBank under the accession no. AQGR00000000. The version described in this paper is the first version, accession no. AQGR01000000.
